# ‘You decided I am old enough for the transition, but not old enough to have a say?’: Exploring young people’s, parents’, and healthcare providers’ views and experiences of Type 1 Diabetes paediatric to adult healthcare transition in Saudi Arabia

**DOI:** 10.1371/journal.pone.0335347

**Published:** 2025-10-29

**Authors:** Nada Aljohani, Angus Forbes, Ghufran Daqrashawi, Mette Due-Christensen, Sara Donetto, Mariam Asaad, Vicki Tsianakas

**Affiliations:** 1 Department of Clinical Research in Diabetes, Division of Care in Long-term Conditions, Florence Nightingale Faculty of Nursing, Midwifery and Palliative Care, King’s College London, London, United Kingdom; 2 Department of Medical and Surgical Nursing, College of Nursing, Princess Nourah bint Abdulrahman University, Riyadh, Saudi Arabia; 3 Saudi Arabian Type 1 Diabetes Association- T1D, Riyadh, Saudi Arabia; 4 National Health Emergency Operation Centre, Ministry of Health, Riyadh, Saudi Arabia; 5 Department of Health Promotion, Steno Diabetes Centre Copenhagen, Region Hovedstaden, Denmark; 6 Department of Medical Education, Brighton and Sussex Medical School, Brighton, United Kingdom; 7 Division of Methodologies, Florence Nightingale Faculty of Nursing, Midwifery and Palliative Care, King’s College London, London, United Kingdom; Levine Children's Hospital, UNITED STATES OF AMERICA

## Abstract

**Background:**

Young people with Type 1 diabetes in Saudi Arabia transition from paediatric to adult care at a culturally defined age of 14, which is younger than the average transition age in Western societies. The aim of this study was to elicit the experiences of young people with Type 1 diabetes, their parents, and healthcare providers in Saudi Arabia as they transitioned from paediatric to adult care.

**Methods:**

In healthcare, Design Thinking is a human-centred approach that draws on participants’ experiences and perspectives to design and develop interventions, models, or services that meet the needs of stakeholders. This study reports the first inspiration phase of the Design Thinking process. Four parallel exploration workshops were held with pre- and post-transition young people with Type 1 diabetes (n = 12), their parents (n = 8), and healthcare providers (n = 7).

**Findings:**

Six key themes were identified from the workshops’ data analysis. For young people, the key themes were facing the unknown and preparedness; developing autonomy and recognition as an independent person; and interacting with the adult healthcare team. For parents, the themes were navigating the shift in parental role and involvement in care, interacting with healthcare professionals, and changing support needs. For healthcare providers, the key theme was balancing independence and care approaches.

**Conclusion:**

The Inspiration phase of the Design Thinking approach provided valuable insights from the healthcare transition experiences of young people with Type 1 diabetes, their parents, and healthcare providers in Saudi Arabia. The generated insights facilitated the identification of areas for interventions in the process’s following phases.

## Background

The transition from paediatric to adult healthcare services is a significant milestone for young people with chronic conditions or disabilities. One of the most prevalent chronic conditions in adolescence is type 1 diabetes mellitus (T1DM). Saudi Arabia has one of the highest incidence rates for T1DM at 31.4 per 100,000 people, with more than 3,800 new onset cases in people <14 years per annum [1]. Young people with T1DM are typically cared for within a child-centred care model, until they transition to adult diabetes services between the ages of 16 and 18 [2]. The transition to adult care poses significant challenges for young people with T1DM, and it has been estimated that up to 30% of young adults with T1DM disengage with diabetes services during this period [3]. The incidence of diabetic ketoacidosis (DKA) also increases during the transition period leading to an increased risk of hospitalisation [4,5]. As a result, this period can significantly affect long-term diabetes care engagement and outcomes. Actively encouraging young people to engage in their care during this phase may help reduce the risk of future diabetes complications and support their physical, mental, and social well-being [6]. A systematic review of international healthcare transition interventions found that most interventions primarily targeted the structural and organisational aspects of the transition, such as service navigation and scheduling and focus on clinical outcomes, rather than psychological outcomes such as diabetes adaption, acceptance, and self-management activation [7]. As a result, many interventions do not fully address the complex personal, emotional, and psychological needs of young people transitioning from paediatric to adult healthcare systems.

Adolescence is an intense period of physical, emotional, and social development. While this stage of life is a universal experience, it is also culturally bound with variations in the way adolescence is understood and shaped. An example of cultural variation is the length of adolescence which can be influenced by sociocultural traditions [8]. In Saudi, the age of legal responsibility is 15 years [9]. Saudi is an Islamic nation; hence adolescence is also determined by the concept of *takleef* which identifies puberty as the point of transition from childhood to adulthood, in religious terms. This is reflected in the age of transition to adult healthcare in Saudi, which is at 14 years of age, when the young person will automatically transfer to adult care without transitional management [10,11]. Therefore, the transition from paediatric to adult is much earlier than other countries which is typically at the age of eighteen or older [2,12]. As a result, there is a little or no transitional care provision in Saudi for young people with T1DM [13], and research into the experiences of young people with T1DM, their parents, and healthcare providers (HCPs) on the transition in Middle East and North Africa (MENA) region is very limited. Therefore, this study aimed to elicit the views and experiences of adolescents with T1DM, their parents, and paediatric and adult healthcare providers regarding the transition from paediatric to adult diabetes care in Saudi.

## Methods

This study was conducted as part of a co-design study involving young people, parents, and HCPs to co-design a paediatric-to-adult T1DM healthcare transition model in Saudi. The data presented in this study relate to the first phase of the co-design process which involved eliciting the experiences of young people, parents, and HCPs of the transition.

### Study design

This study uses a co-design approach which is called Design Thinking [14]. Design Thinking is a human-centred approach that engages users throughout the design process and uses their experiences and ideas to develop innovative healthcare services and interventions [15]. In healthcare, design thinking has been used to enhance the experiences and outcomes of service users, facilitate innovation, and improve healthcare processes efficiency [16]. The Design Thinking process consists of three iterative phases [17]:

- **Phase 1.** ‘inspiration’, this involves exploring the physical, social, and emotional needs of the participants to gain a deep understanding of their experiences and challenges.
- **Phase 2.** ‘ideation’, brings stakeholders together to develop and test ideas that could serve as potential solutions to the problems identified in the inspiration phase.
- **Phase 3**. ‘implementation’ phase, prototypes generated in the ideation phase are iteratively evaluated and refined to enhance their effectiveness.

This paper presents the outcomes of the inspiration phase.

The study was designed in consultation with a Patient and Public Involvement (PPI) group. Key recommendations from this group were:

- The activities should be mixed sex, scheduled on a weekday evening, and held in a private, neutral location not associated with the participating diabetes centre or university.
- Almost all young participants expressed a preference for attending the research activities independently, without their parents present.

### Sampling, screening and recruitment

Groups of 12–16 young people, 6–8 parents, and 6–8 HCPs were recruited purposively, young people and parents included those who were pre and post transition. The sample was recruited from a tertiary diabetes centre in Riyadh, Saudi Arabia Which serves over 15,000 people with T1DM. The centre is one of the largest in the country, it operates multiple specialised diabetes clinics, offering services in paediatric diabetes, diabetes complications, diabetes technology and gestational diabetes.

#### Inclusion criteria.

Young people with T1DM were eligible if they have been diagnosed at least one year prior to recruitment and had undergone or would undergo a transition from paediatric to adult healthcare between March 2019 and March 2023. This timeframe was selected to reduce recall bias. Young people had the option to participate in the study independently or alongside their parents, and similarly, parents could participate regardless of their child’s involvement. Parents were included if they had a young person with T1DM who experienced or would experience healthcare transition within the specified timeframe. HCPs included adult and paediatric endocrinologists involved in transitional care (at least one encounter with a young person with T1DM transitioning), and included: diabetologists, diabetes specialist nurses, diabetes educators, dietitians, psychologists, and social workers.

#### Exclusion criteria.

Young people with additional chronic comorbidities or cognitive or learning difficulties were not eligible. Parents with multiple children with T1DM and prior experience with the transition outside the study’s defined timeframe were also excluded.

#### Recruitment.

HCPs at the centre contacted potential participants to seek their permission to be contacted by NA. Multiple strategies were used to recruit young people, parents, and HCPs within the service; These included: promotional leaflets; contacting local support groups; and using X (formerly Twitter). Recruitment materials contained: the study title and an overview of the objectives in lay terms; participation criteria; an outline of the benefits and risks of participation; the time commitment involved; financial compensation for participating; and the researchers’ contact details. Recruitment period started on 1^st^, March 2023 and ended on 31^st^, May 2023. Prior to providing written informed consent, potential participants were sent electronic or printed pictorial information leaflets outlining the study steps and what would be expected of them. Each group of participants was provided with materials that were appropriate for their age and level of health literacy [18]. A dual consenting process was employed to obtain approval first from the parents and subsequently from the young person. The researcher collected demographic and self-reported clinical information during the consenting process. The clinical data focused on information relevant to the transition, such as the duration of diabetes, insulin delivery mode, continuous glucose monitor use. In addition, young people’s self-reported capacity to manage diabetes daily (collected using a tick box question of ‘being able by myself’, ‘being able with parental support’, or ‘dependent on parents’) was collected. The participants were repeatedly reminded that they could withdraw their contributions at any time before October 1^st^, 2023 (the date after which the data were anonymised), but none of them did.

### Ethical approval

Ethical approval for the study was granted from King’s College London Research Ethics Committee (Project Reference: HR/DP-22/23–34655), Princess Nourah bint Abdulrahman University’s Institutional Board Review (Reference: 22–0653), and King Saud University’s Institutional Board Review (Reference: 23/0143/IRB).

### Data collection

Data were collected over six months between 1^st^, June and 31^st^, August 2023. Four parallel exploratory workshops were held: one for young people who were anticipating the transition, another for young people who had already transitioned, a third for both pre- and post-transition parents, and a fourth for HCPs from paediatric and adult care. The workshops were facilitated by 2 researchers and youth worker, they rehearsed the workshops content prior to data collection (See S1 File). The workshops were held in a private, easy access, informal venue away from clinical settings [19]. NA, GD, and MA led the workshops, a youth worker was also present at the workshops for young people, they facilitated warm-up activities and supported engagement and participation. Several strategies were employed to maximise engagement and enhance group dynamics, these included: warm-up activities and games; collaboratively setting ground rules; emphasising that everyone’s opinion was valid; utilising a Mentimeter; and allowing time for individuals to think and write down thoughts on sticky notes prior to the group discussion [20]. At the end of each workshop, NA and MA debriefed each group and elicited their feedback to improve future workshops.

#### Phase 1 of Design Thinking: Inspiration (exploration workshops).

**Fig 1:**
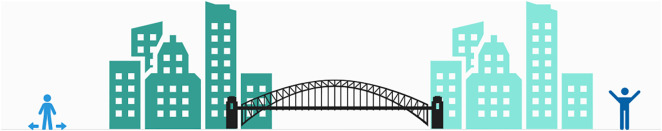
Healthcare transition journey map.

**Fig 2:**
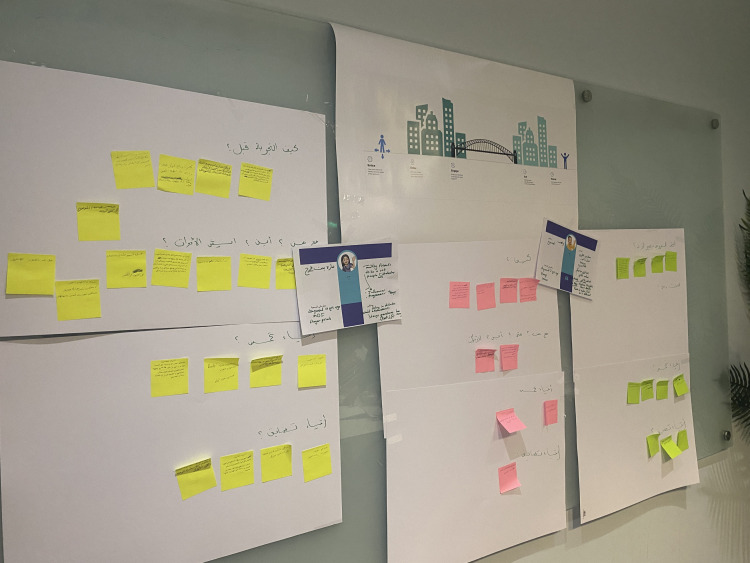
Healthcare transition journey map in workshops. The layout of the journey map with the fictional characters cards in the workshops’ space.

The Design Thinking inspiration phase involved in-depth exploration and questioning of both idealised and actual experiences regarding the transition from paediatric to adult diabetes care. The main goal was to identify key moments and interactions that reflected participants’ perspectives and transition experiences. The sequence of these interactions over time and space is known as the patient’s journey [21]. The idealised experiences of young people and parents who were anticipating the transition, were elicited using the ‘path of expression’ method which began with them envisioning their service, communicating ideas to the group, visualising an ideal experience, and then participating in developing solutions based on the idealised experiences [22]. Young people and parents who had already gone through the transition in addition to the HCPs exploration workshops reflected on existing processes and identified dilemmas and challenges in current care. During this phase, each group used visualisation techniques such as journey mapping, card sorting, and fictional characters. A journey map visually represents the participants’ experiences and interactions with healthcare services [23]. In this study, healthcare transition was seen as an ongoing process and represented as a metaphor for crossing the paediatric-to-adult healthcare bridge (See Fig 1). The map also included design thinking prompts like what, how, when, and where, as well as motivation and demotivation. The prompts were used to help participants through the process of identifying their experience key moments and interactions and placing them on the journey map. Card sorting was used to organise and prioritise key moments in the transition experience. Fictional characters were also used as discussion tools to allow participants to share their experiences and thoughts in the third person voice [14,22]. Two characters (male and female) were printed on large cards, with fill-in descriptions that included: name, age, diabetes story, health details, things that excite me, and things that make me feel down. The young people collectively decided on the characters’ descriptions. MA placed these cards at the centre of the journey map to help each group visualise the characters moving through the transition phases. Figs 1 and 2.

A bridge crossing metaphor was used in the journey map to help participants visualise the transition as a dynamic process.

#### Exploration workshops.

Two workshops were divided were held with young people: one with those expecting the transition (aged 12–14 years); the other with young people who had previously transitioned (aged 15–17 years). Participants varied in terms of age, gender, duration of T1DM, and healthcare transition exposure. The workshops lasted three hours. Each stage of the paediatric-to-adult healthcare transition process was assessed independently by the young people, with the flexibility to recognise any potential overlap between phases. The participants were encouraged to independently reflect on their own experiences within each stage or to envision the feelings, ideas, or behaviours of others (using the fictional characters). The participants were asked to write their thoughts on sticky notes, which they then placed on the journey map.

The parents’ exploration workshop followed the same format with introductions, warm-up activities, and a collaborative setting of ground rules and aims. The workshop lasted three hours, with the discussion focused on the same journey map used in the young people’s workshops, adapted to reflect the parents’ experiences and perspectives.

The exploration workshop with the HCPs was conducted virtually via Zoom and lasted two hours, to accommodate their demanding schedules. NA facilitated the session and used a digital version of the journey map using Zoom’s whiteboards. The workshop began with brief instructions, ice breakers, and then a demonstration and practice on how to edit and use the virtual journey map.

### Data analysis

**Fig 3:**
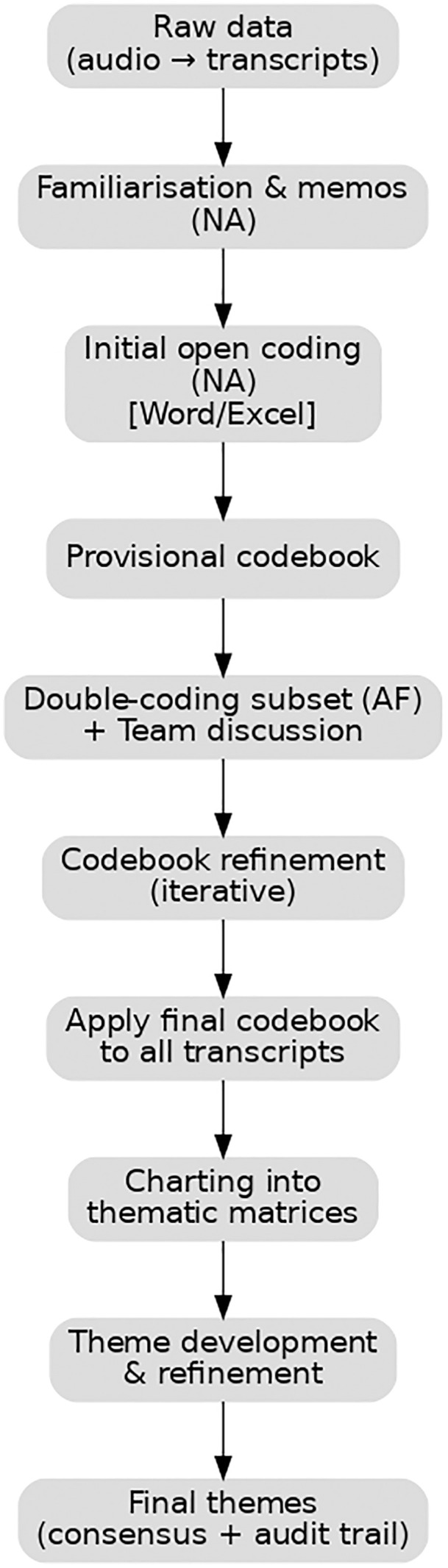
Thematic data analysis.

NA transcribed the workshop recordings translating the core content of the dataset from Arabic to English. Framework Analysis was used to analyse the transcripts inductively and deductively approach, this approach allows the integration of a priori constructs such as those identified in the literature review and for new themes to emerge [24]. The analysis was conducted following steps of Framework Analysis: familiarisation, thematic framework identification, indexing, charting, mapping, and interpretation. Reading the transcripts, journey map postings, and observational notes from the workshop enabled NA to become fully immersed in the data. The key moments and interactions identified in the workshops by young people, their parents, and HCPs were mapped over the overlapping phases of the transition journey, from which a preliminary coding framework was created. Data were analysed using an iterative thematic approach. Transcripts were read repeatedly for familiarisation and analytic memos were written (NA). NA performed line-by-line open coding using Microsoft Word/Excel (codes recorded in a dedicated spreadsheet with source, line number and exemplar quotation). Initial codes were grouped and a provisional codebook with definitions and exemplar quotations was developed. A purposive subset of transcripts was independently double coded by AF to check consistency; discrepancies were discussed in regular team meetings (NA, AF, VT, MDC, MA, GD) and the codebook was iteratively refined. The final codebook was applied to the full dataset and coded extracts were charted into thematic matrices (Excel) to organise data by theme, participant group and workshop. These matrices were interrogated to identify patterns, connections and higher-order themes; disagreements were resolved by consensus. Analytic memos and an audit trail of coding decisions were kept throughout [25]. Fig 3 demonstrate the process of this analysis.

A flowchart of the thematic data analysis steps showing how data moved from transcription and familiarisation through open coding, codebook development, independent double-coding and team consensus, and final charting into thematic matrices to derive higher-order themes.

Thematic matrices were created by rearranging the data according to a thematic framework that combined both a priori and inductively derived constructs. The a priori constructs were drawn from the study objectives and sensitizing concepts in Design Thinking (e.g., user needs, pain points, and transition touchpoints), as well as from existing literature on paediatric-to-adult transition [7]. These provided an initial structure to guide coding, while the framework was iteratively refined to incorporate emergent themes arising directly from participants’ accounts. The framework was refined iteratively through the renaming of existing themes and the identification of additional themes. In the final interpretive phase of framework analysis, participants’ accounts of their views and experiences were synthesised to develop a conceptual understanding of their transition experiences.

### Reflexivity and trustworthiness

To address the potential for subjectivity and bias in the development of the framework, the researchers used a team-based approach to develop the framework, involving multiple researchers with different perspectives and expertise. This helped ensure the framework was comprehensive and reflected the data’s diversity. The methodological approach to this study provided constant opportunities for member checking and iterative clarification of generated input and feedback during workshops. NA maintained a reflective journal to document methodological choices and their justifications, alongside her personal reflections on beliefs and insights [26].

## Findings

### Participants’ characteristics

A total of twenty-seven participants were recruited, including six young people with T1DM at the pre-transition stage and six who had already transitioned to adult services, eight parents, and seven paediatrics and adult HCPs. Out of the twelve participating young people, seven were males, of these 6 were pre-transition and 6 were post-transition. For the pre-transition young people workshop, the median age was 12.5 years, and the median duration of diabetes was 2.5 years. The median age for young people in the post-transition workshop was 15.5 years, and the median duration of diabetes was 4.5 years. Notably, only women (mothers) took part in the parent’s workshop. The HCPs group comprised one adult endocrinologist, one paediatric diabetologist, one dietician, two certified diabetes educators, and two diabetes specialist nurses. Of all groups, eighty-six percent were Saudi Arabian nationals (see Tables 1–3).

**Table 1:** Participating young people.

Pseudonym	Age (years)	Duration of diabetes (years)	Transition status
**Jameel**	12	1	**Pre-transition**
**Sara**	13	11	
**Yasser**	14	10	
**Lana**	12	2	
**Ahmed**	12	3	
**Rayan**	13	1	
**Mona**	16	7	**Post-transition**
**Sama**	15	2	
**Rana**	16	7	
**Kareem**	15	4	
**Ismaeel**	14	3	
**Turki**	16	5	

**Table 2:** Participating parents.

Pseudonym	Role	Transition status
**Muneera**	Mother	**Pre-transition**
**Amal**		
**Nora**		
**Hind**		
**Safiya**		**Post-transition**
**Amnah**		
**Azza**		
**Fauzia**		

**Table 3:** Participating healthcare providers.

Pseudonym	Role
**Reema**	Diabetes specialist nurse
**Sahar**	Diabetes specialist nurse
**Hamad**	Diabetologist
**Fatimah**	Certified diabetes educator and dietician
**Lama**	Certified diabetes educator and health education specialist
**Sultan**	Adult endocrinologist
**Hashim**	Paediatric endocrinologist

### Themes

Six broad themes were identified from the data analysis, three were related to young people, two themes related to parents, and one theme to HCPs. These themes and subthemes are described below and illustrated with selected quotes. Table 4 summarises the six themes and subthemes.

**Table 4:** Key themes and subthemes from the young people, parents, and HCPs’ exploration workshops.

Participants	Key theme	Subthemes
***Young people***	**Facing the Unknown and Preparedness**	• Feeling unprepared • Navigating gaps in diabetes education
	**Developing Autonomy and Recognition as an Independent Person**	• Seeking greater autonomy • Being acknowledged as an independent person
	**Interacting with the Adult Healthcare Team**	• Expectations and preferences for interactions with adult healthcare • Impersonal and disconnected adult care
***Parents***	**Navigating the Shift in Parental Role and Involvement in Care**	• Coping with the loss of caregiving role • Feeling left out and not being heard
	**Interacting with Healthcare Professionals and Changing Support Needs**	• Impact of Healthcare providers’ communication style and attitude • Paediatric care is no longer suitable
*HCPs*	**Balancing Independence and Care Approaches**	• Balancing needs and expectations. • Paediatric and adult care transition contrasts

#### Experiences and views of the young people.

The pre-transition group discussed their previous experiences in paediatric care as well as their worries and concerns about the anticipated transition to adult care. The post-transition group reflected on their experience and the impact of the transition on their lives.

## Theme 1: Facing the unknown and preparedness

Uncertainty and feeling unprepared for the transition resonated strongly in discussions with both groups of young people. Their feelings of uncertainty were associated with not feeling prepared, and a lack of educational support. Young people discussed preparedness in two categories: organisational preparedness, which includes understanding how, when, and what will happen during the transition, its impact on them; and personal preparedness, which involves feeling confident in their ability to self-manage their condition.

### Subtheme 1a: Feeling unprepared

Many of the young people expressed a strong desire to better understand the transition process and its potential impact on their lives, as they often felt unprepared and uncertain about what to expect. The young people who had already transitioned described it as being a distressing time in their lives.

*‘I did not understand anything. I was fourteen, and it was my last appointment at the paediatric clinic. I went in as a child. Then they (the paediatric team) said, ‘Off you go, your next appointment will be in the adult clinic.’ Just like that, it made me feel like an item being carried between different places.’* (**Turki**, 16 y/o, post-transition)

Post-transition young people recalled the initial moment they were informed about the transition by their paediatric HCPs or parents. This moment of transition came as a surprise to many leaving little time for preparation or support.

*‘If this policy existed since before..., why was it so last minute? You would not move schools, jobs, or leave your house without a plan or without directions. So why is this any different?’* (**Kareem**, 15 y/o, post-transition)

Young people who had yet to transition also expressed the importance of understanding what their transition would entail. A significant challenge in understanding and preparing for the transition was the uncertainty stemming from not knowing what to expect.

*‘I am afraid of not knowing what to do... I do not like feeling this way.’* (**Rayan**, 13 y/o pre-transition)

Young people approached the transition with concerns and anxiety about being left uninformed and without guidance. They also expressed their desire to understand and to prepare for the transition.

*‘I just wish that they (the paediatric team) would not let me go without making sure that I understood what to do in the transition.’* (**Sara**, 13 y/o pre-transition)

The post-transition young people shared how a lack of information left them feeling disconnected and frustrated throughout the transition. Some felt unsupported and abandoned by their paediatric HCPs. They shared that they felt overwhelmed and lost after navigating the transition period. Their transition was challenging both emotionally and socially, extending beyond the change in their care providers.

*‘I did not understand anything. I just followed my mother, and I remember that she was confused about the system of the adult clinic. We barely got to the first appointment, and I fell behind in school, you know, because of all that stress added to dealing with the diabetes.’* (**Ismaeel**, 14 y/o, post-transition)

In retrospect, many of the post-transition young people wished that they had received more support and guidance. They felt rushed and pressured by the rapid pace of the transition process.

*‘I wanted to shout and say, please pause for a minute and ask, Now I have to do what?’* (**Rana**, 16 y/o, post-transition)

Both pre- and post-transition young people experienced uncertainty as a result of inadequate transition preparation, leaving them feeling overwhelmed and disconnected from their healthcare transition.

### Subtheme 1b: Navigating gaps in diabetes education

All pre-transition young people agreed that diabetes education was necessary to feel prepared for the transition and improve their self-management skills. Pre-transition young people were concerned about managing their diabetes independently after the transition. Some mentioned that they relied solely on their mothers for guidance, as their paediatric HCPs had never included them in discussions about their treatment plans.

*‘They [paediatric HCPs] spoke quickly with my mother and gave her instructions. Honestly, I did not listen... I never did because their words were always directed at her. She would tell me what was important later.’* (**Yasser**, 14 y/o, pre-transition)

*‘I want to learn from the paediatric clinic and prepare as much as possible before I transition to the adult doctor.’* (**Ahmad**, 12 y/o, pre-transition)

Those who were more recently diagnosed felt they did not know enough about how to manage their diabetes for the transition.

*‘I am not used to having diabetes yet. I do not fully understand it. I want to learn and understand more. The adult clinic will expect me to know already.’* (**Rayan**, 13 y/o, pre-transition)

Some post-transition young people reported receiving education directly from their adult healthcare provider for the first time, through one-to-one education-focused appointments or group-based classes. They also sought clinical advice from follow-up appointments and contacted designated helplines to troubleshoot managing blood glucose levels, insulin correction doses, and using diabetes technology. Young people had positive experiences when the adult providers provided relevant diabetes-related education. It made the young people make feel understood and engaged with their adult care.

*‘The adult clinic provided a good opportunity for me to learn more about my diabetes. We had a monthly online group lecture. Sometimes it had a specific topic, and sometimes it was an open discussion…. It was online, so it was easy to access from anywhere. I liked the open discussion because I could ask questions…. I liked the ones [topics] about food and diabetes management tips if it were something practical, I could try it.’* (**Sama**, 15 y/o, post-transition)

Other young people learnt about diabetes self-management from informal sources like peers with T1DM, support groups, and T1DM-specific social media pages.

*‘There is always something to learn about managing diabetes. Other people who are just like me share their experiences on Twitter and Instagram, and it has been helpful to me to follow and learn more.’* (**Mona**, 16 y/o, post-transition)

Young people needed diabetes education to prepare and help them navigate their transition and to enable independent self-management before and after the transition.

## Theme 2: Developing autonomy and recognition as an adult

During adolescence, young people are forming their identity as adults and seeking recognition of this identity by others. This change impacted their transition experiences and relationships with their families (there mothers in particular) as they sought greater autonomy in their diabetes self-management and clinical follow-up responsibilities. Regardless of their healthcare transition status, both groups of young people were still navigating these challenges.

### Subtheme 2a: Seeking greater autonomy

Post-transition, young people preferred to attend their clinic appointments independently. Some reported that they had been attending independently since the age of fifteen and felt comfortable attending on their own. Other young people continued to attend with their mothers, despite preferring not to. When asked why they had attended with their mothers, they explained that it was a difficult conversation, and they wanted to spare their mothers’ feelings.

*‘My mother has been taking good care of me. It does not feel right to me to tell her I want to attend my follow-ups on my own.’* (**Ismaeel**, 14 y/o, post-transition)

The post-transition group emphasised that their parents were still dealing with the shock and did not want to burden them further. Similarly, the pre-transition young people wanted to openly express their desire for independence but were cautious, fearing that doing so might be perceived as unappreciative and may upset their mothers.

*‘My mother worries too much about me, and I love her for that, but we agreed that I should start to attend the clinic on my own just to prove that I am capable of managing. It went well so far, and I think it helped her, too. She is way less anxious.’* (**Turki**, 16 y/o, post-transition)

All the participating young people anticipating the transition preferred that their mothers, rather than HCPs, deliver the news and explain the transition to them, highlighting the level of involvement of mothers in the care and responsibility of their children’s diabetes. However, the preferences of young people who had already transitioned were divided, with some preferring the mother and others preferring the paediatric healthcare providers. The following quotes demonstrated these divergent perspectives:

*‘The doctor of the house…Mama!’* (**Jameel**, 12 y/o, pre-transition)

*‘Maybe the mother mentions it first at home, but then the paediatric professional should explain why and how with full details.*’ (**Yasser**, 14 y/o, pre-transition)

### Subtheme 2b: Being acknowledged as an independent person

Young people anticipating the transition expressed their concerns about being treated in a patronising manner, with their parents or paediatric healthcare providers attempting to conceal information or avoid providing explanations to shield them from distress. They desired a clear understanding of the purpose of the transition and its potential impact on them.

*‘Just because I am young and have a condition does not mean that I am weak and cannot understand or decide what is right for me. I am the person living with diabetes every day.’* (**Rayan**, 13 y/o, pre-transition)

A post-transition young person described a distressing situation when their paediatric healthcare providers asked them to leave the room to discuss their transition with their parents.

*‘When the physician wanted to inform my mother about the transition, he asked me to leave the room … I felt so small and helpless. Why this way? I can listen... I can understand… Give me the chance. You decided I am old enough for the transition, but not old enough to listen to the news and have a say?’* (**Kareem**, 15 y/o, post-transition)

Other young people described similar experiences in which, despite having transitioned, they felt excluded from their own healthcare. For young people in the pre-transition phase, this confirms their concerns about the lack of information and explanation, which they associate with feeling excluded.

## Theme 3: Interacting with the adult healthcare team

Young people discussed their perspectives and experiences regarding clinical follow-up with adult healthcare providers following the transition. A major contrast between the groups was that pre-transition young people were concerned about the complexity of the clinical guidance they might receive and expected to be overwhelmed by its intensity and quantity. The post-transition young people’s experience showed an overall decline in healthcare provider support after transition, attributable mostly to the pre-existing difference between paediatric and adult care models and service organisation, but also to a variety of factors such as degree of familiarity between the young person and their HCPs, providers’ time capacity, and the young people’s ability to advocate for themselves. As these young people transitioned from paediatric to adult care and became more independent, they wanted more time and support to gain the confidence necessary to voice their concerns and negotiate the complexities of the adult clinic setting.

### Subtheme 3a: Expectations and preferences around interactions with adult healthcare

Pre-transition young people discussed their expectations and preferences of adult care, they emphasised the importance of wanting to be welcomed and understood by adult HCPs as they were experiencing a major change, not merely attending another routine clinic appointment.

*‘They [adult HCPs] should explain to me. Initially, they could welcome me and not speak quickly, allowing me to speak and express my concerns.’* (**Sara**, 13 y/o, pre-transition)

Young people also felt strongly about having adequate space and time to speak up, ask questions, and raise concerns without being interrupted or rushed.

*‘Diabetes is a 24-hour condition. You cannot explain everything at once, keep things simple. Also, when I am in the clinic, I do not want to feel that I am in the way of the next patient … No rush!’* (**Ahmed**, 12 y/o, pre-transition)

Young people were concerned about the use of negative demotivating language, which could discourage them and make them want to withdraw from care.

*‘I am afraid that the adult doctor will expect more from me... that things will be complicated... I would be discouraged when they tell me, ‘You failed’ if targets were not met for any reason.’* (**Yasser**, 14 y/o, pre-transition)

Pre-transition young people had certain expectations and preferences around their adult care. Their views primarily focused on how they wanted to be spoken to in an engaging way that recognised their transition.

### Subtheme 3b: Impersonal and disconnected adult care

The post-transition young people frequently stated that their initial impression of adult HCP’s behaviour towards them when they transitioned was disconnected. They did not feel comfortable or confident asking questions or raising concerns.

*‘I was with my paediatric team for five years before I transitioned. They knew everything about me and my family. I have been with the adult team for two years now. It is so hard to have that kind of familiarity and connection. I tried, but they seem busy or not interested.’* (**Mona**, 16 y/o, post-transition)

Most post-transition young people said their clinical follow-up consisted of routine management, prescriptions, diabetes screening and booking follow-up appointments.

*‘To be honest, it is just about renewing my prescription now. Why bother? It is always the same. Your A1C is so and so, and you need to do this and that’* (**Turki**, 16 y/o, post-transition)

*‘When I first transitioned, the adult HCPs were very technical. All they wanted to address was A1C, make sure you book your next follow-up appointments, and so on. I expected to be asked how the transition was... How are you managing that change? Can we make it better? But none of that.’* (**Sama**, 15 y/o, post-transition)

Some post-transition young people admitted to dreading follow-up appointments in the adult clinic and frequently skipped them for reasons such as being judged or exposed to blameful language about diabetes-related complications. Few mentioned that these negative interactions had either improved or no longer bothered them. Such impersonal interactions from adult care made the young people feel disconnected and hopeless about their adult care.

#### Experiences and views of parents.

## Theme 4: Navigating the shift in parental role and involvement in care

Parallel to the young person’s healthcare transition, the parents and young people are also transitioning: the transfer of self-management and clinical follow-up responsibility from the parents to the young person. Mothers associate this transition with their identity as caregivers of a young person with T1DM. While post-transition mothers shared their emotional experience of having to relinquish this role, pre-transition mothers expressed concern about this upcoming transition.

### Subtheme 4a: Coping with the loss of caregiving role

Post-transition mothers expressed feelings of loss and sadness about being less involved in their child’s care. They reflected on how their identity is shifting as their children gain independence. They shared that, since their child’s diagnosis, their entire lives had revolved primarily around their child’s condition.

*‘I am so isolated now... I feel that I have to entrust my son to God, but at the same time, I’m afraid. He doesn’t talk to me or ask for my opinion about anything related to diabetes. He says, “I manage my affairs, and everything is fine, with a glycated haemoglobin level of 6% or 7%, and I’m happy. So, it’s not your responsibility.” I felt completely isolated from my role as a mother. Who am I if not the mother of a child with diabetes?’* (**Amnah**, post-transition mother)

Two of the pre-transition mothers gained insight while listening to post-transition mothers share their experiences. Until this discussion, they had not realised that eventually they would be transferring the diabetes self-management responsibility to their children as they become autonomous adults. They expressed their thoughts as:

*‘What do they mean? (referring to other mothers) What do you mean when you say you do not attend the clinic with them anymore?? She is my child... even if she is an older person. Even if I have to carry her all the way... she is my life.’* (**Hind**, pre-transition mother)

*‘The problem is also that, as a mother, I see my child, even if they’re fourteen years old, as a child. But others see them as grown-ups; they come and tell him, “You’re a man.” So, the hardest thing is when you tell them, “Okay, go ahead and act on your own.” This was perhaps the hardest thing.’* (**Nora**, pre-transition mother)

Those experiences highlighted an often-overlooked aspect of the transition that parents face as they navigate the shift in their caregiving role.

### Subtheme 4b: Feeling left out and not being heard

Following further exploration into what the transition would mean for the mothers, one major concern was that they would become less involved in their child’s healthcare planning and decision-making and felt excluded. The mothers felt their children were too young and lacked the capacity for full independence and emphasised that they need to be heard. They also had reservations about entirely trusting the new adult clinic. The post-transition mothers reported instances in which their child’s care plan underwent significant changes (e.g., insulin types and doses) without them being aware.

*‘For me, I’m always with my daughter, but if she has to go on her own, and sometimes I might be travelling, and her father is busy, so she has to go alone, and she comes back with strange things!... New medications without consulting any of us!’* (**Fawziah**, post-transition mothers)

*‘I feel that nobody will tell me anything anymore. But if there are any changes, it’s better for them to go back to the mother and clarify, especially since I may not have experience with the new plan.’* (**Azza**, post-transition)

This view was echoed by pre-transition mothers, who already felt overlooked and disregarded by paediatric HCPs, even while being present in the clinic with their children, and they worried this would be worse in the adult clinic.

*‘I used to ask the doctor, “Is my son’s height normal? Is his weight normal? Are his nerves normal?” I know that even if he’s eleven years old... twelve years old... we need to examine the nerves, and he says no! We will examine them after he is twenty years old. But my son is suffering now!’* (**Amal**, pre-transition mother)

*‘My daughter sometimes wakes up crying from the pain in her legs, saying, “Mom, I can’t feel my legs,” which means there’s a problem! Why do you always force me to live in tension? Why don’t you try to listen to mothers and do something at least to ease our minds?... Listen, even if it’s not supported by medical evidence, if it’s something simple and not costly, just do it.’* (**Safiyah**, pre-transition mother)

## Theme 5: Interacting with healthcare professionals and changing support needs

Pre- and post-transition mothers expressed their perceptions and previous experiences with adult healthcare providers. They talked about the adult providers’ communication, tone, and attitude. Mothers also discussed the potential positive impact of the transition to adult care, if it focussed on supporting young people’s overall development.

### Subtheme 5a: Impact of Healthcare providers’ communication style and attitude

The mothers’ views about HCPs primarily revolved around their communication style and behaviour. Pre-transition mothers expressed worries that interactions with adult HCPs would be more impersonal and lack the emotional bonds and understanding that their children experienced in paediatric care.

*‘What I fear the most is that the adult doctor himself might not be suitable and might not understand children. Do you know what I mean? I mean, the cold treatment. This fear... even the fear that they might argue with me… blame me. I mean, it’s enough that I am constantly blamed by the people around me, I don’t need this. So, it’s the way of delivering the information and whether the doctor will be good. Will he cooperate with me? Will he really be of help to me or not?’* (**Nora**, pre-transition mother)

Some mothers felt that this change in communication just reflected that adult HCPS communications are adult focussed and not nuanced to young people.

*‘The way information is delivered is because the adult team will consider children as grown-ups, so they will convey the information in a—let’s say—a bit dry or formal manner because they’re used to dealing with adults, so their approach can be dry. This is scary. Or that they put more blame on the parents because people might hold parents more accountable. This is the scariest thing about transitioning.’* (**Muneera**, pre-transition mother)

The experiences of post-transition mothers aligned with these expectations, as they relayed how their children’s first experiences with adult care were ‘traumatic’ and discouraging. The adult teams in these cases focused exclusively on treatment changes and preventing diabetes complications. They found their tone to be judgmental and sometimes blameful.

*‘The adult care approach was very, very dry and very difficult. He came and told my son, “You have diabetes; you must adhere to the treatments so that you don’t develop complications, and if you do not, then your situation is not good.” It was traumatic!’* (**Azza**, post-transition mother)

All mothers highlighted the need for psychological support for both parents and young people to help manage the stress, anxiety, and other emotional challenges during the transition.

Pre-transition mothers were worried that the transition would be particularly difficult for young people with shorter diabetes duration. They were concerned that adult care might overestimated their understanding and self-management ability and fail to adjust for this or provide additional support.

*‘Another potential downside is that I am worried that adult providers might think that my child already knows and is experienced with diabetes, so providers might just want to provide prescriptions and deal with administrative processes. Nothing more.’* (**Nora**, pre-transition mother)

### Subtheme 5b: Paediatric care is no longer suitable

Although pre-transition mothers were anxious about their children’s transition to adult care, they recognised that it was unavoidable and that continuing under paediatric care may no longer be the best approach as their children mature into adolescence.

*‘Dealing with a paediatrician in general or a paediatric endocrinologist is different from dealing with an adult doctor, I mean, they still treat them as children. This can annoy the children themselves, you know, because they feel they are older, at an advanced stage, and more aware. This can bother them. Saying things like “come on, champ”, can make them want to say, “We have grown up, we need someone to treat us differently?”’* (**Amal**, pre-transition mother)

Another aspect to consider is the onset of puberty, particularly in females. Mothers believed that the transition to adult care would be more appropriate following the menarche, although they were also concerned about its impact on their daughters’ interactions with HCPs.

*‘Puberty is a critical period, and girls do not want the providers to physically examine them. I mean, girls even feel shy about the doctor, which is natural. We have our culture and our religion. If one day she felt exposed and she did not want to be examined, it would be a distressing experience for her.’* (**Fawziah**, post-transition mother)

This opinion was echoed by the post-transition mothers who shared experiences where the adult team provided support and addressed sensitive topics, such as sexual functioning and diabetes, which is considered a taboo subject in Arab culture. The mothers expressed some shock at this but also their relief, as they were not adequately equipped to deal with such topics.

*‘My son went to the adult clinic once, and because he is grown up, the doctor asked him about the sexual aspect, something I never thought about. So, he asked me to step outside so he could discuss it with him. This was something I never imagined thinking about or discussing with my son, and indeed, diabetes affects everything. So, he was really happy when he left that appointment.’* (**Amnah**, post-transition mother)

*‘I remember when my son went to adult care for the first time. The doctor examined my son and asked him about his genitals and private areas in general. Honestly, I was shocked by the question; my imagination had not gone that far yet. Could it be possible that he was already experiencing problems there? The doctor told me, “Yes, it is possible.”’* (**Azza**, post-transition mother)

#### Experiences and views of healthcare providers.

## Theme 6: Balancing independence and care approaches

Both paediatric and adult HCPs discussed the multifaceted healthcare transition and its challenges. They recognised the need to equally support the variable needs of a young person with T1DM and their parents, in addition to addressing their expectations around the transition and adult healthcare. Throughout the workshop, there were some contrasting opinions between the paediatric and adult healthcare models. Paediatric healthcare is often family centred, while the adult healthcare model typically prioritises promoting independence. These fundamental differences in goals, treatment strategies, and young people’s needs led to varied perspectives and discussions among the participants.

### Subtheme 6a: Balancing needs and expectations

Based on their experiences, HCPs recognised the impact and challenges for young people during the transition. The insights from HCPs included concerns about how to prepare the young person and ensure that they gain more autonomy in managing their condition, specifically the transition of self-management and clinical follow-up responsibilities from the parents to the young person with T1DM. Adult HCPs believed that excessive parental interference prevented young people from developing confidence and autonomy, by their continued attendance at clinic appointments into adulthood which limits their ability to address the young person directly when discussing their care.

*‘One issue that we frequently encounter is that mothers continue to attend appointments with their children even when they are eighteen and twenty years old. We keep asking them not to. We explain that our goal is to encourage the young person to become independent and manage their own affairs. It takes time and effort to explain why and convince the mothers.’* (**Hamad**, adult diabetologist)

*‘I agree that it is difficult to address someone directly in the clinic while their mother is present and constantly interrupting. It is almost never about what the young person truly needs from that appointment, but rather about the mother’s needs, who understandably require some support as well. It’s just too difficult to have two agendas in one appointment.’* (**Sultan**, adult endocrinologist)

In contrast, paediatric HCPs considered family presence and involvement to be important during the transition to ensure the engagement of the entire family, particularly both parents in the process.

*‘I believe that the mother’s presence provides a great opportunity to engage the entire diabetes-affected family. Actually, it should be both parents. The young person will receive more support, and the entire family can go through this process together. More so for a child of separated or divorced parents, we always have a challenging time with these children because they live in two separate environments, and if one parent knows how to support their child, the other typically does not.’* (**Lama**, certified diabetes educator)

### Subtheme 6b: Paediatric and adult care transition contrasts

Another contrast between paediatric and adult HCPs, was in relation to the young person’s knowledge about their condition, and their confidence in self-management. The adult HCPs were very focussed on aspects of self-management such as carbohydrate counting and insulin administration following transition to adult care.

*‘The first thing to be done in the adult clinic is to review and start a structured diabetes education plan as soon as possible so there will not be any gaps that could adversely affect the young person’s diabetes outcomes.’* (**Lama**, certified diabetes educator)

Conversely some of the paediatric providers considered that doing this immediately after transfer to be overwhelming. They felt that most young people are too young to fully grasp these concepts independently of their parents when they transition. Diabetes educators had differing perspectives they wanted the young people to be fully prepared by providing them with a comprehensive education before the transition.

*‘In my experience with young people, I think rushing with diabetes education as soon as possible can be overwhelming for the young person it should take a while. It might put them off the adult clinic. They just went through a major change in their lives, probably the biggest since they were diagnosed.’* (**Fatimah**, paediatric dietitian)

## Discussion

Similar to the experiences reported of young people with T1DM worldwide, this study confirmed that transition for young people with T1DM in Saudi from paediatric to adult healthcare is a complex and challenging period. However, the transition for young people in Saudi is unique as it occurs at the culturally defined cut-off age of 14 for adulthood, which is younger compared to Western societies. Hence, transitional care in the Saudi population needs to incorporate these sociocultural differences, as with other Gulf Cooperation Council (GCC) states [27].

Current international and Saudi clinical recommendations emphasise the importance of proactive planning for the transition process, both young people and parents [28–31]. Our study has revealed that these recommendations are under considered in Saudi, leading to significant levels of uncertainty, and a lack of preparation and readiness for the transition. A similar lack of preparation has been reported in other studies with most transition initiatives emphasising the transfer of care form paediatric to adult services, rather than the transition as a developmental phase impacting young people and their families [32,33]. In our study, the young people wanted to keep the frequency of adult care follow-ups similar to those in paediatric care, to support them and extend the process without making it feel abrupt and overwhelming. Similar experiences were reported in other countries, for example, the United States (US) [29]. Previous studies have defined the transition as a period of emotional disruption and that young people without support lack the mental processes to regulate the feelings and anxieties it can generate [34,35]. Given the additional burden of diabetes in their lives, navigating these changes with T1DM can be very challenging. Young people can be given the time they require to anticipate and process these changes if HCPs developed plans that address the young person’s individual physical and emotional readiness.

Solowiejczyk [36] defines T1DM as a ‘family disease’ because the focus of management should be on the family and not exclusively on the person with diabetes. However, adolescence can also be a period of increased tension among young people with T1DM relationships and their parents, as parents can feel anxious about relinquishing and ceding control to their child [37]. These concur in observations of this study where the dynamics between the young person and their parents, and the young person and their HCPs were evident. As these dynamics were under considered the young people and mothers felt inadequately prepared and supported to develop their new roles and responsibilities. The study findings also show that HCPs find it difficult to speak directly to the young person about their care and assess their independence and needs while the mother was present during or after the transition. This challenge had a negative impact on young people because they felt unheard and, despite being transitioned to adult care, were not treated like adults. In some instances where the HCPs successfully attempted to address the young person directly, the parents often felt unsupported and excluded. Mothers felt a sense of loss when they had to relinquish their role as carers, but they also expressed concerns about adult care being intimidating and disconnected and leaving their children unsupported. Making decisions about the level of parental involvement was frequently discussed among young people, their parents, and HCPs. All the participants agreed that this was a sensitive topic and that they needed support to initiate and maintain open discussions, as well as to make decisions about setting and agreeing on boundaries. Similar to our study, a US study found that over 60% out of 27 parent participants did not discuss the transition with their children [38]. Therefore, parents need greater support throughout the transition so they can understand the need for the young person to become more autonomous.

The young people in this study expressed a strong need for diabetes education that was tailored in both content and delivery. They acknowledged their unique differences, particularly their varying levels of competency and ability to adjust to the changes occurring during this period. They sought a diabetes education that addressed these specific needs. Young people, parents, and HCPs emphasised the need for clear information and education to support young people and their parents navigate the uncertainties of the transition. However, this education was under provided and there is a need to incorporate a programme of education in the transition that enables young people to set their own agendas, goals, and priorities as well as provide diabetes specific education.

Adolescent speciality healthcare is not yet established in the Saudi healthcare system, and specialists with expertise in managing diabetes during adolescence are scarce. In our study, as young people grew older, parents expressed concerns that paediatric care would eventually become unsuitable for their needs. They also recognised that the current adult HCPs lack the necessary skills to provide care for this specific population, which is similar to other GCC countries healthcare systems [27]. In line with international findings, young people with T1DM in Saudi perceived adult care to be authoritarian and didactic [39]. They also experienced transitional difficulties such as abrupt change, the need to adapt to self-management, and dissatisfaction with the care offered by an adult-oriented model [3].

The findings from the HCPs showed an awareness of the transition, they outlined challenges in relation to; the young person’s readiness for the transition; managing clinical follow-up processes; transition referrals; and providing young people and their parents with clear information about scheduling and attending follow-up appointments. They also highlighted a lack of providers with skills to offer psychosocial support and resources within their organisation.

While this study focused on the inspiration phase of the Design Thinking process, it is important to consider how insights generated at this stage can inform subsequent phases. The ideation phase builds on these insights to generate and prototype possible solutions, while the implementation phase involves testing, refining, and scaling interventions in real-world contexts. The current paper reports only on the inspiration phase, but subsequent work has progressed to the ideation and prototyping phases, which will be reported separately. In the context of paediatric-to-adult transition in T1DM, for example, the empathy and needs mapping conducted during the inspiration phase can lead to the development of tailored preparation materials, services pathways, educational tools, or peer support models that address identified gaps. Beyond T1DM, similar approaches could be valuable in other long-term conditions where the paediatric-to-adult care transition is critical, such as kidney transplantation, congenital heart disease, or cystic fibrosis. By iteratively moving from inspiration to ideation and implementation, Design Thinking offers a structured yet flexible framework to co-create interventions that are both user-centred and adaptable across diverse healthcare settings and contexts.

## Strengths and limitations

To our knowledge, this is the first study to qualitatively explore the healthcare transition for T1DM in Saudi. This study’s findings may be applicable to other GCC countries and generally Middle Eastern countries with similar cultural, religious, and socioeconomic characteristics. This study addresses a significant gap in the literature by contributing valuable insights into the lived experiences of young people and parents managing T1DM in this context. The exploration workshops provided valuable insights into young people, parents, and HCPs’ experiences with the healthcare transition in Saudi. Identifying the key moments and interactions that young people, parents, and HCPs face during the transition was an important step in identifying areas and opportunities for potential solutions in later phases of our larger study. The study findings may also be transferable other GCC countries because of context similarities as discussed earlier.

Other strengths to the study were the engagement techniques used to support and enable all the young people and parents to share their ideas with the group. Young people chose to stay in the room for most of the breaks, to continue conversing with each other and the facilitators. They also expressed their wishes for the research to continue for a longer duration as for most of them it was the first time they met a peer their age with T1DM. The workshops set up gave them a sense of community and belonging.

The study limitations are reflective of all studies of this type in that the views and experiences presented are derivative from the context from which they were yielded. This study was conducted in Riyadh, Saudi’s capital city, where the healthcare system is more developed and structured than in other cities or rural areas of the country. Hence, the experiences of young people in those areas may be different. Nevertheless, it is likely that the overall impressions gained would connect with young people in all areas of Saudi as they are bound in the same transition definition on age. A further limitation was the time available for the workshops, longer sessions with increased frequency and more participants could have provided more time for the exploration process, but funding and logistical constraints limited the number of young people’s and parents’ workshops. The HCPs’ workshop duration was limited to accommodate their busy and demanding schedules. However, despite these limitations the study yielded important data on the experiences of the transitions particularly from the young people and parents.

## Implications for future research and practice

While there is consensus in the existing international recommendations on the importance and benefits of transition practices, there is a lack of theory-informed and evidence-based transition interventions for adolescents with T1DM [40]. Difficulties in standardising the intervention design, due to variations in context and organisational policies indicate the need for adaptive models in the development of new interventions [41]. The findings of this study have explicated the importance of addressing challenges in the provision of transitional support within the Saudi context and healthcare system.

When providing care to people with diabetes, it is important to consider their cultural context. Interventions and practices must be tailored to the psychological and sociocultural needs of young people, their parents, and HCPs, rather than focusing solely on biophysiological outcomes. Providing targeted education for young people’s and their parents’ is important if their experiences are to be improved. It is also clear that HCPs need training how to provide more age-appropriate communication and care planning and engagement for young people and their parents to enhance the impact and quality of the care provided in this transitional phase.

## Conclusion

This study has provided important insights and a better understanding of the challenges that young people with T1DM, their parents, and healthcare providers face when transitioning from paediatric to adult care. Young people felt uncertain and unprepared for the transition. Parents struggled with coping with the shift in their roles. Moreover, it was equally challenging for both young people and parents to navigate the adult care service. HCPs emphasised how the transition service should be organised, however, they did not address the interpersonal dynamics between paediatric and adult teams. The healthcare transition is a journey that requires anticipation, preparation, and support along the way.

## Supporting information

S1 File Exploration workshop plan. (PDF)
