# Supplementary material for: ‘You decided I am old enough for the transition, but not old enough to have a say?’: Exploring young people’s, parents’, and healthcare providers’ views and experiences of Type 1 Diabetes paediatric to adult healthcare transition in Saudi Arabia
Source: PLoS One. 2025 Oct 29;20(10):e0335347. doi: 10.1371/journal.pone.0335347 (PMC12571242; doi:10.1371/journal.pone.0335347)
Supplement: S1 File — (PDF) [file pone.0335347.s001.pdf]


Parents Exploration Workshop Plan

17:00 – Set Up

Nada and Mariam

- A/C
- Projector and Laptop – check Mentimeter.
- Set out snacks and drinks.
- Flip chart and stand.
- Set out chairs.
- Have iPads ready (medical history and voucher details forms).
- Have warm-up activities print-outs ready.
- Have journey map and Post Its ready.

18:00 – Welcome and Thank You (5 mins)

Nada: Welcome and thank you for being here. Name badges are provided. Introduce the concept of doodling and/or taking notes if they have ideas.

Nada, Mariam, Ghufran, and Hanan: introduce ourselves briefly.

Nada – Housekeeping

• Toilets

• Fire exit and procedure if fire alarm (no practice alarms planned)

• Wi-Fi details (write on Flip Chart)

• Phones: On silent; Only for use with Mentimeter and in breaks

• Room (106.1) – If anyone needs some privacy or time out during the group activities

18:05 – Ground Rules (10 mins)

Nada: Introduce the idea of ground rules and why they are important. Explain how to use the Mentimeter (slide 1). Facilitate discussion of the 'ground rules'

Mariam: will be operating the laptop and presenting slides.

Ghufran: write keywords from the discussion on Flip Chart Paper and stick on wall where visible.

18:15 – Introductions (20 mins)

Nada

1. Remind everyone (including researchers/facilitators) to wear their name badges.

2. Question and answer: Start the session with a question-and-answer session, where participants can ask any questions, they may have about the focus group.

3. Introduce Mentimeter (slides 2&3) questions: A. Hobbies/interests; B. One thing that you'd really love to do in the future (that you haven't done yet).

Everyone (including researchers/facilitators) post answers. (Mariam operates Mentimeter) Nada feed-back to group one question at a time (10 mins).

4. Divide the group into two subgroups (3 if the number is odd) and introduce and facilitate the next activity, collaborative storytelling. Nada will prompt each group with a word presented on (slide 4); one person from each group will begin with a sentence containing the word; other group members will each follow and come up with a sentence to form a cohesive story on the spot.

18:35 – Aims (10 mins)

Nada

Set out her aims for the group (Ghufran lists these on Flip chart paper):

If already transitioned:

A. To understand more about the experiences of young people who have T1DM when they transitioned from paediatric to adult healthcare. (Or parents or HCPs)
B. To have a better idea of things that might be helpful for other young people with T1DM who start will transition in the future. (Or parents or HCPs)

If not yet:

To understand more about young people with T1DM's anticipatory experiences and perspectives on their upcoming transition from paediatric to adult healthcare. (Or parents)

Everyone to have a fun day.

Ask young people what they want to get out of the day/ what their expectations are – they can write these on post-its, and researcher/facilitator feed back to the group.

Participants to be given the opportunity to comment on/ edit the researcher's aims and suggest additions (Ghufran add to the Flip chart paper)

Tick box placed next to each item on list, and Flip chart paper to be stuck on the wall so visible throughout the day (when an aim is achieved, the corresponding box will be ticked).

Nada: Before the break, ask if participants have any questions.

18:45 – Break (15 mins)

Prayer space, snacks and drinks are available.

19:00 – Warm Up (10 mins) (30 circles only for parents)

Mariam:

Interduce the rules to 30 circles challenge. Give each participant one of the 30 Circles worksheets. Ask them to turn as many of the circles as possible into a recognisable object in 3 minutes. Give the example of a sun or a smiling face, but no other instructions. After 3 minutes, Nada will compare results! Ask how many people filled in 10, 15, 20 or more circles? Ask for some examples. Did participants use patterns (for example, multiple sports balls)? Did anyone "break the rules" and combine circles? Any similarities between the participants? Or unique objects?

19:10 – Inspiration (45 mins)

Journey Map (Ghufran hang the journey map on the wall)

Nada:

Introduce idea of a journey map, and brief description of the different stages: preparing for

The move to adult care; first time at the adult service; 'settling into' adult healthcare.

Introduce idea of ***fictional characters*** –ask participants to give names and details to these characters (one boy, one girl).

Nada:

- Give everyone *PINK* post it notes.
- Ask questions pertaining to preparing for the move to adult care: (refer to questions in the topic guide specific to each participants group).
- Young people, parents, or HCPS to write their ideas on post it notes and position on the journey map.
- Nada to ask about the post it notes and their position on the map (use '5 whys').
- Mariam and Ghufran facilitate discussion and take notes.

Nada:

- Give everyone GREEN post it notes.
- Ask questions pertaining to first time at the adult service: (refer to questions in the topic guide specific to each participants group).
- Mariam to ask about the post it notes and their position on the map (use '5 whys').
- Nada and Ghufran facilitate discussion and take notes.

Nada:

- Give everyone YELLOW post it notes.

- Ask questions pertaining to ‘settling into’ adult healthcare: (refer to questions in the topic guide specific to each participants group).
- Nada to ask about the post it notes and their position on the map (use ‘5 whys’).
- Ghufran will write participants’ definitions of ‘settling in’ on its section on the journey map.
- Mariam facilitates discussion and take notes.

Nada summarises journey map so far.

19:55 – Wrap up the day and agreement on the next steps.
